# Supplementary material for: Empirical methods for controlling false positives and estimating confidence in ChIP-Seq peaks
Source: BMC Bioinformatics. 2008 Dec 5;9:523. doi: 10.1186/1471-2105-9-523 (PMC2628906; doi:10.1186/1471-2105-9-523)
Supplement: Additional File 1 — A variety of html documents from the USeq web site detailing the available applications, their best usage, output file type descriptions, command line menus, etc. [file 1471-2105-9-523-S1.zip › USeqUserGuides/JavaDoc.zip!/JavaDoc/gata/main/class-use/GATAPlotter.html]

Uses of Class gata.main.GATAPlotter


|  |  |  |
| --- | --- | --- |
| **Overview** \| **Package** \| **Class** \| **Use** \| **Tree** \| **Deprecated** \| **Index** \| **Help** | |  |
| PREV   NEXT | **FRAMES**    **NO FRAMES**     **All Classes** |  |


---


## **Uses of Class gata.main.GATAPlotter**

| Packages that use GATAPlotter | |
| --- | --- |
| **gata.main** | Main app, utility, and params for GATAPlotter. |

Uses of GATAPlotter in gata.main

| Methods in gata.main that return GATAPlotter | |
| --- | --- |
| `GATAPlotter` | `GATAParams.getGataPlotter()` |

| Methods in gata.main with parameters of type GATAPlotter | |
| --- | --- |
| `void` | `GATAParams.setGataPlotter(GATAPlotter gata)` |

---


|  |  |  |
| --- | --- | --- |
| **Overview** \| **Package** \| **Class** \| **Use** \| **Tree** \| **Deprecated** \| **Index** \| **Help** | |  |
| PREV   NEXT | **FRAMES**    **NO FRAMES**     **All Classes** |  |


---
